# Riding the rapids: COVID‐19, the three rivers curriculum, and the experiences of the University of Pittsburgh School of Medicine

**DOI:** 10.1096/fba.2020-00099

**Published:** 2021-02-24

**Authors:** D. Michael Elnicki, Peter Drain, Greg Null, Jason Rosenstock, Ann Thompson

**Affiliations:** ^1^ University of Pittsburgh School of Medicine Pittsburgh PA USA

**Keywords:** Covid‐19 pandemic, curricular adaptation, curriculum reform, pedagogy, remote and hybrid curriculum, safety, teaching models

## Abstract

When faced with the COVID‐19 pandemic this past spring, the University of Pittsburgh's School of Medicine (UPSOM) took rapid steps to ensure the safety of students, staff, and the faculty as well as to maintain the educational process. Curriculum reform efforts, already underway, proved to be an advantage in the transformation. We quickly converted to a remote and then a hybrid curriculum. Research labs were reopened with appropriate safety measures. Clinical experiences for students restarted via a phased process that emphasized safety and graduation requirements. A variety of assessment mechanisms were restarted with appropriate modifications. New teaching models, such as flipped classrooms, have become the norm, and it seems hard to imagine our returning to our old pedagogy. The curriculum committee met continually to guide the process of change and reopening. The curricular adaptation process remains ongoing, and challenges remain. Nonetheless, we have learned from our experiences and hope to use this knowledge gained as we move forward.

## 1 INTRODUCTION

As the COVID‐19 virus swept across the United States, the city of Pittsburgh and University of Pittsburgh School of Medicine (UPSOM) were fortunate in several respects. First, we were not among the first cities hit by the virus, giving us some time to plan. Pennsylvania did not announce a COVID‐19 case until March 6, and the first local case was announced on March 13, which was the day that Governor Wolf shut down the state's schools.^1^ Second, at the medical school, we were already in the process of implementing a curricular reform that emphasized many of the actions we would soon be forced to take. Third, local case numbers never became unmanageably high, as our civic and hospital systems’ leaders took rapid, preventive actions. Elective procedures were cancelled, and outpatient clinics were closed, but health care workers remained prepared. These and other factors enabled the UPSOM to engage in planning with crisis, but not catastrophic, conditions.

In terms of the number of COVID‐19 cases seen locally, our county's peak new case day in the early months was April 3 with 73 new cases. On June 17, there were no new cases, but after a gradual opening of group activities, cases surged locally and peaked county wide at 322 on July 13. Since then, there has been a gradual decline in cases, and there was no surge with the return of the county's 85,000 college students. However, as in the rest of the country, COVID‐19 has disproportionately struck minority communities. Our black population is 13% of the county but has 25% of cases and 17% of deaths.^1^

In the following pages, we reflect on our response to the COVID‐19 pandemic with respect to the functioning of the medical school. Part of the teaching and training involved adaptations that were our best guesses at solving surprising problems imposed or afforded by the COVID constraints. Adapting sometimes led to solutions that we could not have imagined doing pre‐COVID and might be worth keeping post‐COVID. Other adaptations were clearly stopgaps that will be jettisoned along with remnants from aged parts of the curriculum. COVID forced us to move in new ways and provided lessons on faculty, staff, and medical student creativity, flexibility, and resourcefulness to innovate the medical curriculum. Throughout the process, our primary goals were to protect the safety of our students, patients, staff, and faculty as well as to guide students toward completion of the curriculum with coordination by the curriculum committee and the Office of the Dean.

## 2 FRAMING EMERGENT SOLUTIONS

As the pandemic unfolded, UPSOM needed to gather its students and reconsider its curriculum. The curriculum committee began to prepare for COVID‐19 on March 2. Throughout the ensuing months, the curriculum committee continued to meet as scheduled, but remotely as of March 16. Votes were taken to change some graduation requirements, add remote electives, and adopt recommendations from its groups. Its Curriculum Continuous Quality Improvement Subcommittee began a review of the curricular response. Even during the pandemic, the usual business of monitoring the curriculum continued, with reports from students, courses, and the completion of Phase 1 of curriculum reform. Attendance at meetings by members and guests increased due to the ease of remote access. It was suggested by many that these meetings continue remote for convenience of the group.

On March 13, following a university‐level decision, medical students were dismissed from classroom and clinical activities. Most pre‐clinical courses continued virtually through pre‐recorded lectures from prior years. The pre‐clinical students completed their current courses. They were able to complete exams, and they then went on spring break. Not knowing the extent or duration of the pandemic, we took the opportunity of this critical moment to convert rapidly to a virtual curriculum. A transformation in conceptualizing the curriculum needed to happen. Demands for technological support increased rapidly and massively. We knew, based on the experiences of others, that this would be a stressful time for all stakeholders.^2^ Consequently, mindfulness and wellness for students, staff, and faculty needed to be factored into planning.

Upon return from spring break on March 30, students were able to view synchronous or asynchronous lectures. Students attended small groups via Teams or Zoom. In‐person clinical activities for pre‐clinical students were cancelled or postponed. Remote electives were designed by course directors and approved by the curriculum committee. The sustainability of such efforts is an important point to consider, and it is important to understand the effort needed to transition, to maintain, and to deliver the curriculum.

On March 17, the Association of American Medical Colleges (AAMC) advised that clinical students be removed from direct patient contact. On March 24, it became obvious that the suspension would continue through April. The students were able to finish courses with case‐based learning and didactics, both of which could be done remotely. Virtual electives for fourth year students were quickly developed and approved by the curriculum committee. A virtual anesthesia clerkship was constructed. Still, the fundamental question of how to make up patient contacts remained. The curriculum committee voted to create a COVID‐19 Clinical Transition Task Force on April 1 with 30 days to plan the restart. We agreed that time on task did not equate to competency. With the recommendations from the Task Force, the Committee voted on May 5 to make the following changes for the restart:

1. Rather than taking both the Family Medicine and Adult Outpatient Medicine Clerkships, students needed to choose an outpatient medicine selective. This action shortened the time necessary to meet graduation requirements and added flexibility to the clinical schedule in anticipation that clinical sites would be closed during COVID‐19 surges.
2. A surgical subspecialty was added to the last two weeks of the Surgery Clerkship, instead of as a separate clerkship, which further shortened required clinical weeks.
3. Students began Adult Inpatient Medicine, Surgery and Acting Internships on May 18, and all clerkships were running by June 8.
4. We established clinical site reactivation requirements, such as Personal Protective Equipment (PPE) and clinical case mix. The latter had changed considerably at some sites after the pandemic unfolded. Medical students were prohibited from caring for COVID patients or persons under investigation for COVID‐19 for reasons of safety and per AAMC guidelines.

The suspension of clinical activities for students created some notable issues with clinical experiences. The first priority was to ensure that all eligible medical students graduated on‐time. After that, the plan was to ensure continuity within the curriculum for rising fourth year students and the new‐to‐the‐wards third year students. This included a review of program objectives and clinical experiences via the Learning Logs system. Graduation requirements were reviewed and changed if necessary. Some of these changes included dropping graduation year elective requirements from eight to seven, extending the deadline for USMLE Step 1 completion until the end of 2020, case‐by‐case early conferral of the MD degree, and waiving the USMLE Step 2CS requirement. This requirement was replaced by the Clinical Skills Assessment, our home grown version of Step 2 CS. Students who could not make up the anesthesiology clerkship had the opportunity to take an elective or selective. Due to the risk involved in anesthesiology, the required clinical procedures were reviewed. The curriculum committee approved remote or simulated experience for bag/mask ventilation and endotracheal intubation. Pre‐clerkship courses that require clinical opportunities were delayed or modified. Advanced physical exam was forced to end early in March, which means a delay of some instruction until spring 2021. The clinical experiences course saw a restructure of some requirements that included a book club and discussion group.

Communication, at first, hardly kept up with the barrage of changes. Twice weekly updates from the Office of Medical Education began in March and offered students, staff, and faculty, the most up‐to‐date information. Students, staff, and faculty appreciated the frank information they contained as the plan for restart and the continuation of remote learning pushed into summer. The updates proved to be particularly valuable to keep up with new initiatives. For example, the emails helped us to disseminate information on our social medicine initiatives during the unrest of early summer. Comments from surveys have asked that such emails continue after COVID‐19.

It became apparent that our home‐grown administrative software (AMP‐UP), inadequate pre‐COVID, was now a system that was not nimble enough to bend with the flexibility required to continue our mission during this pandemic. The COVID‐19 clinical transition task force was unable to begin the proper planning of the clinical restart due to the system's inability to change the length of courses beyond four‐week and eight‐week offerings. The task force was unable to bend the system toward a common best practice, shortening the clerkships. The system also failed in the scheduling of courses for students, creating frustration for students who were unsure of their own schedules at times until the day before the course began. In light of a planned curriculum reform, we concluded that a new system is needed to grow a flexible and resilient curriculum.

## 3 INTERVENTIONS WORTH SHARING

When faced with the conditions imposed by the pandemic, our Dean's Office and curriculum committee had to move quickly and decisively. Zoom and Microsoft Teams were made available to all students in preparation for the shift to distance learning. Teaching support services were made available to instructors and students to ease the transition. A well‐developed student advising system already existed and was shifted to on‐line. Our admissions process was rapidly converted to online interviews and committee meetings.

Initially, all activities were forced to be fully remote, but we felt that some student experiences needed to remain in person. We embraced the principles and best practice guidelines of our university's overall approach to hybrid curricula, combining remote and in‐person learning. In‐person activities, masked and socially distanced, were reserved for only those activities essential to completing the course learning objectives. An example is anatomy lab, long a site of excellence in our medical school. The cadaver lab was restructured and students rescheduled by the course director to allow for socially distanced participation. Other lab experiences were switched to distance formats through multi‐media hardware, such as internet‐enabled cameras for bird's‐eye views of prosections. Software such as the Complete Anatomy platform was used by both in‐person and remote learners.

For other courses, such as Evidence‐Based Medicine (EBM) Foundations and EBM Applied, COVID‐19 was the catalyst for ongoing changes. In the EBM Foundation course, for example, lecture module materials with formative quizzes in previous years had already been uploaded as module chunks to our learning management system. The formative assessments are forwarded to the facilitators so that concepts and skills that were challenging could be further elucidated and practice in subsequent small group workshops no longer in person but now using Teams online. The strategy to have the pre‐class formative assessments forwarded to facilitators to shape and make more effective and efficient the in‐class segments of the educational cycle moves these flipped‐style formats towards smart‐flipped workshops, which engage such cognitive priming and focusing assessments in the pre‐class segment to shape the subsequent in‐class segment. For EBM Foundations, the COVID‐19 adaptation was simply to transition the initial Introductory class, and the in‐person final exam to remote versions, using Zoom and Examsoft, respectively.

Still other foundation courses required more drastic stopgaps. Among these were to replace in‐person lectures by recording and posting them as a pre‐workshop segment followed by an online synchronous segment. We learned our previously recorded podcast lectures often sorely needed a step‐up in instructional design, content, and delivery based on student evaluations. We recognize that person to person teaching is still the backbone of medical education. Therefore, in‐class online workshops were designed to focus on learning concept and skill application and reflection, often in the setting of published or de‐identified cases from our health system.

Courses providing introductions to clinical aspects of medicine switched to the use of role play and to standardized patients, often using videoconferencing. Our medical school trains and uses a substantial pool of standardized patients, and we did not want them to be idled or endangered during the pandemic. Because all of our students complete longitudinal research projects, reopening research laboratories to students was a priority and was quickly achieved. For all students, staff and the faculty, the re‐opening of research across the medical school has been a success of logistics, provision of PPE, testing and training.

The re‐engagement process for clinical rotations proved to be a delicate task. We were accustomed to large and varied services across an entire hospital system. When we were prepared to begin the reintegration process, however, many outpatient and inpatient services had not yet regained pre‐COVID volumes. Many attending physicians were seeing patients in a combination of virtual and in‐person clinics, which made coordinating with students difficult. Others expressed concerns about having students participate in direct patient contact, citing patient, and student safety issues.

As part of the process, prior to beginning each clerkship, students were required first to demonstrate proficiency in PPE relevant to the clinical rotation. Second, the number of students on any given service was limited. For example, only one student could be present at the bedside during rounds. Third, didactic sessions, except for teaching rounds, were conducted virtually. We began with rotations that were critical for students’ graduation, such as acting internships and critical care rotations. Then, we phased in clerkships and clinical electives.

Assessment strategies continued but in somewhat altered forms. Our preceptors’ clinical assessments that were based on direct observation were already online. Subject exams were converted to online. We have long had a rich, standardized patient system for both formative and summative assessments. The standardized patients have been extensively trained and would be difficult to replace. Fortunately, they were retrained to provide virtual scenarios for teaching seminars and objective structured clinical exams (OSCEs).

After reopening traditional clinical sites, we began to reactivate service and volunteer rotations. Some students remained involved during the crisis, in logistic support at our hospitals, as happened in other schools.^3^ Some of our students worked with the Health Department and helped with contact tracing. Others worked in hospital command centers where they helped us to triage messages, did focused histories, and presented them to preceptors. Our students have a long history of service engagement in the community. Many of these rotations provide important services to the poor and underserved, and students were eager to restart them. They include a woman's shelter, an eye service, and free clinics for adults and children. A structured process has been developed, where sites complete a restart application form stating the community need and the presence of faculty support. The form specifically addresses the need for PPE, level of student activity, likelihood of COVID‐19 exposure risk.

## 4 TESTS & PRELIMINARY OUTCOMES OF INTERVENTIONS

These adaptations are ongoing, and formal outcome assessments need to follow. However, we are seeing some positive, early signs of stability and improvement. Students re‐entering basic sciences report high levels of satisfaction. As clerkship students re‐enter clinical sites, preliminary reports from teaching faculty and clerkship directors indicate that students seem adequately prepared for their clinical assignments. Scores on subject exams have remained stable.

We have sought feedback from the affected stakeholders within UPSOM. The sample is small and informal; however, we were able to gain insights as to how our policies were being perceived. These groups have included medical students, administrative staff who deal with medical students on a regular basis and faculty course and clerkship directors. We were relieved to see that all stakeholder groups overwhelmingly felt that they had been protected during the COVID‐19 response since that had been our primary concern. A majority of those responding also felt that the medical school had helped them to adapt to the changes required by the pandemic.

There were, however, some concerns about the curricular transitions. Comments from faculty, staff, and students expressed worry that the transition will lead to issues in future clinical rotations, residency, and beyond. Many felt the quality of their courses suffered in comparison to the previous in‐person educational experience. Faculty members’ comments generally reflected gratitude for support received in this transition, but several felt inadequately supported. Some faculty members noted appreciation for flexibility in how to set up their remote courses. Several faculty members commented that the differences in regulations and policies between the school and the hospital system were a hindrance to their adapting. Small numbers of students report access issues related to software, internet, and reliable hardware and that these challenges limit their ability to interact with the remote curriculum. These issues are particularly concerning given our absolute reliance on the capacity of users to work within a remote environment at this time, and we are addressing them. Other student concerns raised in comments included isolation, financial and technological issues, and uncertainty about the future. Students did not receive any formal training in how to become remote learners, but they did receive “helpful hints” which were posted on an internal website.

## 5 REFLECTIONS

1. The Pittsburgh area is not prone to disasters. We do not get hurricanes, tornados, earthquakes, forest fires and rarely get significant flooding. Although we had a disaster plan, it needed to be dusted off and addressed. We have come away from the COVID‐19 pandemic realizing that we may need to respond rapidly to unforeseen disasters in the future. We have also become more comfortable with collaborations, as groups were forced to work together who had not previously done so.
2. We realized that we will probably never go back to the old ways of education. Prior to COVID, we had begun moving to distanced learning. COVID‐19 catalyzed implementation and designing of our goals for scientific active learning and teaching. Both the faculty and the students have become more comfortable with it and will incorporate distance learning techniques into future versions of courses. We have noted several positive aspects, such as the increased ability for all students at various clinical sites to attend synchronous didactic sessions without needing to travel. Conference attendance at large meetings, like grand rounds, has increased, in part since travel and distance are no longer issues.
3. Lessons learned from this crisis will inform our ongoing curricular reform efforts. We had already begun a major curriculum overhaul prior to the pandemic. We had committed to decreasing in‐person teaching sessions, particularly in the basic sciences. Small group, interactive sessions were already replacing lectures. Flipped classroom models have become the new normal in our curriculum with scientific, active learning and teaching.^4^ Our students prefer collaborating with each other and faculty members. They also appreciate frequent formative assessments. We have noted in other educational programs that co‐generational activities improve the depth of understanding and retention.^5^ The pandemic furthered the move to competency based assessments. We have seen that time on task does not equate to competency. We increased formative and summative assessments to assure competency.
4. An unexpected outcome of the epidemic is our strategic experience offering all our learners simultaneous in‐person and remote conferences, meetings, and workshops. All these sessions are recorded for later viewing. Faculty, staff, and students appreciate the ability to use the style of learning best suited to them. Unexpectedly, COVID‐19 has launched us into developing creative uses of software and hardware for more effective pedagogy. One area of opportunity being used that could be generalizable is smart video‐conferencing hardware to bring together learners and teachers by integrating software.
5. We have been able to switch to online exam formats seamlessly. Examsoft was in place for in‐house exams. The NBME has been extremely cooperative in enabling distanced subject exams. Our OSCEs have been transformed to virtual versions.
6. Pittsburgh was swept into the Black Lives Matter movement occurring concurrently with the pandemic. Our move to include social determinants of health into the new curriculum accelerated and is already being implemented.

## CONFLICT OF INTEREST

None.

## AUTHOR CONTRIBUTIONS

All authors wrote the manuscript and reviewed the final version.
